# Polygenic risk scores for neuropsychiatric, inflammatory, and cardio‐metabolic traits highlight possible genetic overlap with suicide attempt and treatment‐emergent suicidal ideation

**DOI:** 10.1002/ajmg.b.32891

**Published:** 2022-02-21

**Authors:** Giuseppe Fanelli, Marcus Sokolowski, Danuta Wasserman, European College of Neuropsychopharmacology (ECNP) Network on Suicide Research and Prevention, Siegfried Kasper, Joseph Zohar, Daniel Souery, Stuart Montgomery, Diego Albani, Gianluigi Forloni, Panagiotis Ferentinos, Dan Rujescu, Julien Mendlewicz, Diana De Ronchi, Alessandro Serretti, Chiara Fabbri

**Affiliations:** ^1^ Department of Biomedical and Neuromotor Sciences University of Bologna Bologna Italy; ^2^ Department of Human Genetics Radboud University Medical Center, Donders Institute for Brain, Cognition and Behaviour Nijmegen The Netherlands; ^3^ National Centre for Suicide Research and Prevention of Mental Ill‐Health (NASP) Karolinska Institute Stockholm Sweden; ^4^ Department of Psychiatry and Psychotherapy Medical University Vienna Vienna Austria; ^5^ Department of Psychiatry Sheba Medical Center, Tel Hashomer, and Sackler School of Medicine, Tel Aviv University Tel Hashomer Israel; ^6^ Laboratoire de Psychologie Médicale Université Libre de Bruxelles and Psy Pluriel, Centre Européen de Psychologie Médicale Brussels Belgium; ^7^ Imperial College School of Medicine London UK; ^8^ Laboratory of Biology of Neurodegenerative Disorders Department of Neuroscience, Istituto di Ricerche Farmacologiche Mario Negri IRCCS Milan Italy; ^9^ Department of Psychiatry Athens University Medical School Athens Greece; ^10^ University Clinic for Psychiatry Psychotherapy and Psychosomatic, Martin‐Luther‐University, Halle‐Wittenberg Germany; ^11^ Université Libre de Bruxelles Brussels Belgium; ^12^ Social, Genetic & Developmental Psychiatry Centre Institute of Psychiatry, Psychology & Neuroscience, King's College London London UK

**Keywords:** gene sets, major depressive disorder, polygenic risk scores, suicide, treatment‐worsening/emergent suicidal ideation

## Abstract

Suicide is the second cause of death among youths. Genetics may contribute to suicidal phenotypes and their co‐occurrence in other neuropsychiatric and medical conditions. Our study aimed to investigate the association of polygenic risk scores (PRSs) for 24 neuropsychiatric, inflammatory, and cardio‐metabolic traits/diseases with suicide attempt (SA) or treatment‐worsening/emergent suicidal ideation (TWESI). PRSs were computed based on summary statistics of genome‐wide association studies. Regression analyses were performed between PRSs and SA or TWESI in four clinical cohorts. Results were then meta‐analyzed across samples, including a total of 688 patients with SA (*N*_eff_ = 2,258) and 214 with TWESI (*N*_eff_ = 785). Stratified genetic covariance analyses were performed to investigate functionally cross‐phenotype PRS associations. After Bonferroni correction, PRS for major depressive disorder (MDD) was associated with SA (OR = 1.24; 95% CI = 1.11–1.38; *p* = 1.73 × 10^−4^). Nominal associations were shown between PRSs for coronary artery disease (CAD) (*p* = 4.6 × 10^−3^), loneliness (*p* = .009), or chronic pain (*p* = .016) and SA, PRSs for MDD or CAD and TWESI (*p* = .043 and *p* = .032, respectively). Genetic covariance between MDD and SA was shown in 86 gene sets related to drugs having antisuicidal effects. A higher genetic liability for MDD may underlie a higher SA risk. Further, but milder, possible modulatory factors are genetic risk for loneliness and CAD.

## 1 INTRODUCTION

Suicide is the second most prevalent cause of death among youths, with European countries having the highest death rates (WHO, 2019). It has been estimated that one person every 40 seconds dies by suicide worldwide, resulting in ~800,000 deaths per year. The World Health Organization (WHO) has listed reducing suicide mortality among the stated objectives for 2030, to be achieved by adopting preventive methods and supporting mental health and healthy lifestyles.

Among people dying by suicide, ~40% had previously attempted suicide (Turecki et al., 2019). The lifetime prevalence of suicide attempt (SA), which represents a self‐injurious behavior with some intent to die, is estimated at 2.7% compared to an even higher prevalence of 9.2% for suicidal ideation (SI) (Nock et al., 2008). SI, which spans from thinking about to planning suicide, is frequent in depressive states across a number of psychopathologies, and, in turn, it elevates SA risk by 18% within 12 months of SI occurrence (Borges et al., 2010; Turecki et al., 2019).Overall, suicidal phenotypes (SPs) are 10 times more prevalent in patients with psychiatric conditions than in the general population, with a frequency of mental illness of ~90% among individuals who attempt/die by suicide (Turecki et al., 2019). Common psychiatric comorbidities include mood disorders and schizophrenia, as well as post‐traumatic stress disorder and substance (particularly alcohol and cannabis) use disorders (Arsenault‐Lapierre, Kim, & Turecki, 2004). Moreover, insomnia was shown to predict SA/SI in patients with anxiety or depression (Dolsen, Prather, Lamers, & Penninx, 2020). It is also worth noting that ~10% of individuals with major depressive disorder (MDD) may experience the emergence or worsening of SI in the early stages of antidepressant treatment (Cristancho et al., 2017). As a result, despite a long‐standing scientific debate, the US Food and Drug Administration has retained a black box warning regarding an increased risk of SI among individuals aged <25 who are prescribed antidepressants and still advices close monitoring in all other age groups (Friedman, 2014).

In addition to the occurrence of SPs in the context of psychiatric disorders, SA/SI risk increases more than four times in the presence of somatic multimorbidity (Stickley et al., 2020). Interestingly, suicidal events are often preceded by access to primary care for physical complaints (Luoma, Martin, & Pearson, 2002; Stickley et al., 2020). In particular, individuals with a history of coronary artery disease (CAD) have shown higher SI rates, independent of other comorbid psychical diseases and depression (Moazzami, Dolmatova, & Feurdean, 2018), and patients with SI/SA showed higher burden of overweight/obesity, hypertension, and other cardiovascular diseases than healthy controls (Zhong et al., 2020). Moreover, patients with diabetes mellitus have shown an increased likelihood of SA, and metabolic syndrome has been positively associated with SI (Bolton, Walld, Chateau, Finlayson, & Sareen, 2015). SPs were also related with exposure to common infectious agents and repeated inflammatory insults (Coryell et al., 2020; Isung et al., 2019), while C‐reactive protein (CRP) levels, which are a biomarker of chronic low‐grade inflammation, were associated with higher suicidality among psychiatric patients (Miola et al., 2021). Suicidality has also been predicted by chronic pain conditions and more frequent bouts of intermittent pain (Racine, 2018). However, the indirect psychological effects of somatic diseases on the emergence of SPs are not easily disentangled from the possible presence of biological factors shared with SPs (Ko et al., 2019).

The biopsychosocial model may be a way to tie everything together. This paradigm considers suicidal events as the result of the interplay between distal (or predisposing) factors, consisting of genetic/epigenetic influences and early life adversities leading to lasting alterations in gene expression, and proximal (or precipitating) factors (Turecki et al., 2019). Among proximal factors, perceived loneliness was shown to be important (Calati et al., 2019). Personality traits represent key mediators between distal and proximal factors. In this regard, impulsive‐aggressive traits are among the most associated with suicide and are often found in externalizing disorders like attention‐deficit/hyperactivity disorder (Nock et al., 2009). In addition, anxiety traits/disorders and impaired self‐control have been closely related to the transition from SI to SA (Nock et al., 2009). Other personality traits, such as agreeableness and conscientiousness, have been linked with reduced SI rates, while neuroticism and openness were associated with higher SI, though all these effects were age dependent (Na et al., 2020). Individuals with early‐onset SA exhibited higher levels of neuroticism and lower levels of extroversion than nonsuicidal depressed individuals, while those with late‐onset SA outperformed depressed controls on orderliness, a subcomponent of conscientiousness (Szucs, Szanto, Wright, & Dombrovski, 2020).

Although the biopsychosocial model is helpful in comprehending better the phenomenon, the underlying etiopathogenic mechanisms of SPs are still far from being fully elucidated. Genetic factors have been shown to play a role in SPs, with heritability ranging from 30% to 55% in twin and adoption studies (Fanelli & Serretti, 2019; Voracek & Loibl, 2007). However, the single‐nucleotide polymorphism (SNP)‐based heritability ($h_{\mathrm{SNP}}^{2}$) of SA was only ~4% in genome‐wide association studies (GWAS), but more insights are likely to come as sample sizes increase (Mullins et al., 2019; Ruderfer et al., 2020).

A promising research opportunity is represented by polygenic risk scores (PRSs), which summarize the additive genetic risk conferred by multiple common variants across the genome (Choi & O'Reilly, 2019). As well as being promising tools for stratifying individuals at higher risk, PRSs also allow the investigation of possible shared genetics between complex traits (Fanelli et al., 2021). Therefore, PRSs can provide insights on the genetic overlap between SPs and other psychiatric or physical conditions. Further information may derive from stratifying the genome by functional annotations (Lu et al., 2017), which could lead to a deeper understanding and the formulation of new hypotheses about the biological mechanisms underlying SPs. Current knowledge on the mechanisms of action of medications with well‐established antisuicidal properties may be exploited to prioritize candidate gene sets of interest and investigate, at the pathway level, any genetic sharing between suicidal and other comorbid phenotypes that PRS analyses could reveal (Fanelli et al., 2022). In this regard, the most consistent data on a protective effect on suicidality concern clozapine, ketamine, and lithium, whereas evidence on antidepressants is mixed (Stone et al., 2009; Turecki et al., 2019). Multiple evidence suggests lithium treatment to be more effective than placebo in reducing SPs in mood disorders (Turecki et al., 2019), and lithium levels in drinking water have been inversely associated with suicidality at the population level (Memon et al., 2020). Similarly, clozapine has good efficacy in reducing the risk of recurrent suicidality in patients with schizophrenia spectrum disorders, and ketamine showed a rapid effect in reducing SI in depressed subjects (Turecki et al., 2019).

In order to contribute in filling the discussed gaps in knowledge, this study aimed to (a) test the association between the PRSs for 24 neuropsychiatric, inflammatory, and cardio‐metabolic traits/diseases with SA in major psychiatric disorders or treatment‐worsening/emergent suicidal ideation (TWESI) in depression; and (b) investigate the common molecular pathways underlying cross‐phenotype genetic overlaps by exploring pairwise genetic covariance stratified by candidate gene sets, which were selected as those involved in the response/pharmacodynamics of clozapine, ketamine, and lithium.

## 2 METHODS

### 2.1 Target samples for PRS analyses

#### 2.1.1 Clinical Antipsychotic Trials of Intervention Effectiveness

The Clinical Antipsychotic Trials of Intervention Effectiveness (CATIE) study is a randomized clinical trial sponsored by the National Institute of Mental Health (NIMH) intended to assess the efficacy of one first‐generation and four second‐generation antipsychotic medications in patients with schizophrenia. Eligible patients were initially randomized under double‐blind conditions to perphenazine, olanzapine, quetiapine, risperidone, or ziprasidone and received medication for up to 18 months or before discontinuation of treatment for any cause. A total of 738 individuals were genotyped. Additional information can be found elsewhere (Stroup et al., 2003).

#### 2.1.2 European Group for the Study of Resistant Depression

The Group for the Study of Resistant Depression (GSRD) is a multicenter study designed to investigate antidepressant treatment response/resistance in patients with MDD. Patients were excluded if they were diagnosed with any other primary psychiatric disorder or substance use disorder in the previous 6 months. Treatment with antidepressants was conducted in a naturalistic manner following the best clinical practice (each antidepressant was taken at an appropriate dose for ≥4 weeks during each depressive episode). A total of 1,346 patients were genotyped. Additional details on the study design and population can be found elsewhere (Dold et al., 2018).

#### 2.1.3 Sequenced Treatment Alternatives to Relieve Depression

The Sequenced Treatment Alternatives to Relieve Depression (STAR*D) study was conducted to evaluate the efficacy and tolerability of different antidepressant therapies across four sequential treatment stages in patients with MDD. Patients with nonpsychotic MDD (DSM‐IV criteria) were recruited from primary care or outpatient psychiatric services. Individual‐level genotypes of 1,939 participants were obtained. The study design and population are described in depth elsewhere (Howland, 2008).

#### 2.1.4 Systematic Treatment Enhancement Program for Bipolar Disorder

Systematic Treatment Enhancement Program for Bipolar Disorder (STEP‐BD) was a prospective study aimed at improving bipolar disorder (BD) treatment and management and evaluating the longitudinal outcome of the disorder. A hybrid design was used by STEP‐BD to gather longitudinal data as patients move between naturalistic studies and randomized controlled trials. Patients aged 15 yo with type I or II BD, cyclothymia, BD not otherwise specified, cyclothymia or schizoaffective disorder were enrolled. A total of 955 participants were genotyped. The research design and study population are described in depth elsewhere (Sachs et al., 2003).

### 2.2 Target phenotypes for PRS analyses

Two binary target phenotypes were considered: lifetime suicide attempt (SA), which was extracted from all the four target samples, and treatment‐worsening/emergent suicidal ideation (TWESI), which was available in the two MDD samples (GSRD and STAR*D). SA was defined as an intentional, self‐injurious behavior with some potential for lethality (Dennehy et al., 2011; Marangell et al., 2008). Any information of suicidal events having these characteristics and occurred at any point during the lifetime was collected and considered for analysis. Further details on the extraction of the SA and TWESI phenotypes from each target sample can be found in Supplementary information (paragraph 1 Data S1).

### 2.3 Genotyping and quality control of the PRS target samples

Details on the genotyping of each target sample are provided in Supplementary information (paragraph 2 Data S1). Preimputation quality control (QC) was carried out in all the target samples by first removing monomorphic variants and the SNPs with a genotype missing rate ≥5%. Individuals who had sex mismatches, a genotyping rate <97%, abnormal heterozygosity, high relatedness (identity by descent [IBD] > 0.1875) (Anderson et al., 2010) were excluded. Non‐European individuals were identified and excluded based on self‐report information, and a further check was made by inspecting the principal component (PC) analysis plots. Population PCs were calculated using a linkage disequilibrium‐pruned set of variants (*R* ^2^ < .2), and individuals falling outside ±5 *SD* from the mean of the first 20 population PCs were excluded. Genotype imputation was performed using Minimac3 and the Reference Consortium (HRC) r1.1 2016 reference panel. Variants with minor allele frequency (MAF) <0.01, low imputation accuracy (*r* ^2^ [estimated squared correlation between imputed genotypes and true genotypes] < .30) (Li, Willer, Ding, Scheet, & Abecasis, 2010), and genotype probability <.9 were removed.

### 2.4 Statistical analyses

#### 2.4.1 PRS analyses

PRSs for 24 neuropsychiatric, inflammatory, and cardio‐metabolic traits/diseases were computed as the sum of the number of effect alleles at each SNP position, weighted for their effect size derived from the largest GWAS meta‐analyses available at the time of conducting our analyses. A complete list and further information on base samples is reported in Table 1. To prevent overlap between base and target samples and resulting overfitting in PRS analyses, GWAS leave‐one‐out summary statistics for MDD and schizophrenia (excluding the STAR*D and CATIE samples, respectively) were obtained from the Psychiatric Genomics Consortium.

**TABLE 1:** Base samples used for the computation of the genome‐wide polygenic risk scores

Base trait/disorder	First author	Year	PMID	*N* cases	*N* controls	Total *N*	*N*_eff_
Attention‐deficit/hyperactivity disorder (ADHD)	Demontis	2019	30478444	19,099	34,194	53,293	49,017
Aggression (childhood and early adolescence aggressive behavior)	Pappa	2018	26087016	—	—	18,988	—
Agreeableness	De Moor	2010	21173776	—	—	17,375	—
Alcohol dependence	Walters	2018	30482948	11,569	34,999	46,568	34,780
Alcohol intake (drinks per week)	Liu	2019	30643251	—	—	537,349	—
Anxiety (lifetime anxiety disorder)	Purves	2019	31748690	25,453	58,113	114,019	70,802
Bipolar disorder (BD)	Mullins	2021	34002096	41,917	371,549	413,466	150,670
Coronary artery disease (CAD)	Siewert	2018	30525989	—	—	735,838	—
Cannabis (lifetime cannabis use)	Pasman	2018	30150663	43,380	118,702	162,082	127,079
Chronic pain	Johnston	2019	31194737	—	—	387,649	—
Conscientiousness	De Moor	2010	21173776	—	—	17,375	—
C‐reactive protein (CRP)	Ligthart	2018	30388399	—	—	204,402	—
Extraversion	Van den Berg	2016	26362575	—	—	63,030	—
Insomnia	Jansen	2019	30804565	109,402	277,131	386,533	313,750
Loneliness	Day	2018	29970889	80,134	364,890	445,024	262,818
Major depressive disorder (MDD)	Wray/Howard	2018–2019	29700475–30718901	170,756	329,443	500,199	449,856
Metabolic syndrome (MetS)	Lind	2019	31589552	59,677	231,430	291,107	189,773
Neuroticism	Baselmans	2019	30643256	—	—	523,783	—
Openness to experience	De Moor	2010	21173776	—	—	17,375	—
PGC Cross‐Disorder phenotype	PGC cross‐disorder group	2019	31835028	232,964	494,162	727,126	633,299
Post‐traumatic stress disorder (PTSD)	Nievergelt	2018	DOI: 10.1101/458562	10,643	28,633	39,276	31,036
Schizophrenia (SCZ)	Pardinas	2019	29483656	40,675	64,643	105,318	99,863
Suicide attempt (SA)	Ruderfer	2019	30610202	2,433	334,766	157,366	9,662
Type 2 diabetes mellitus (T2DM)	Mahajan	2018	30297969	74,124	824,006	898,130	272,026

Abbreviations: *N*, sample size; *N*_eff_, effective sample size [*N*_eff_ = 4/(1/(*N* cases) + 1/(*N* controls)].

In each target sample, PRSs for each base trait/disease were calculated using PRSice‐2.2.13 at 8 a priori GWAS P‐thresholds (P_T_) (1 × 10^−4^, 0.001, 0.05, 0.1, 0.2, 0.3, 0.4, 0.5) (Choi & O'Reilly, 2019). SNPs in high linkage disequilibrium were clumped considering a 250 kb window and *r* ^2^ threshold of .1. Logistic regression analyses between PRSs for each base trait/disease and the case–control status (i.e., SA vs. non‐SA, TWESI vs. non‐TWESI) were performed in each target sample, adjusting for population stratification and recruitment centers. Age and sex were not included as covariates in the main analyses in line with previous genomic studies on SA and TWESI (Mullins et al., 2019; Mullins et al., 2014). The proportion of variance in SA or TWESI explained by PRSs in each sample was estimated by Nagelkerke's pseudo‐*R* ^2^ as the difference between the *R* ^2^ of the full model, incorporating the PRS and covariates, and the *R* ^2^ of the null model, including only the covariates. Finally, results of PRSs analyses at each P_T_ across samples were meta‐analyzed for both the target phenotypes using a fixed‐effect inverse‐variance weighted model with the metafor R‐package (https://cran.r-project.org/web/packages/metafor), as done by previous authors (Garcia‐Gonzalez et al., 2017; Zheutlin et al., 2019). Between‐study heterogeneity was assessed using the Cochran's Q test, computed as the weighted sum of the squared differences between the individual and pooled study effects, and Higgin's and Thompson's I^2^, describing the percentage of variation across studies being due to heterogeneity rather than chance (Higgins, Thompson, Deeks, & Altman, 2003). Bonferroni correction was applied to account for the multiple base traits/diseases and the eight P_T_ considered for PRSs analyses (α = .05/[24*8] = 2.6 × 10^−4^).

The statistical power of PRSs was determined via the AVENGEME R‐package (Palla & Dudbridge, 2015). We assumed a covariance between the genetic effects in the base and target samples of 25% or 50% (Garcia‐Gonzalez et al., 2017), while sample and lifetime population prevalences, $h_{\mathrm{SNP}}^{2}$ of base and target phenotypes were derived from the previous literature (Table S1). The PRSs of all the 24 base traits/diseases showed adequate predicting power (>80%) for SA and TWESI.

#### 2.4.2 Genetic covariance analysis stratified by candidate gene sets

To better characterize the biology underlying the genetic overlap of the considered psychiatric and nonpsychiatric traits/diseases with SPs, we performed a pairwise genetic covariance analysis stratified by candidate gene sets using GeNetic cOVariance Analyzer (GNOVA) and GWAS summary statistics as input datasets (Table 1). GNOVA provides genetic covariance estimates robust to sample overlap between the two reference GWASs (Lu et al., 2017). As a starting point, we considered the biological mechanisms involved in the effects of drugs with well‐established antisuicidal effects (i.e., clozapine, ketamine, lithium), as these have strong evidence of being implicated in preventing SPs. We decided not to select gene sets from previous genetic studies on SPs but rather to use a pharmacologically guided strategy, in order to have a larger pool of candidates and keep the focus on pathways that are potentially druggable. We reviewed the previous literature looking for genes and molecular pathways implicated in the pharmacodynamics of these drugs (Table S2). The mechanisms and genes identified by the literature review were used as search queries in the Molecular Signatures Database (MSigDB) v7.2 (https://www.gsea-msigdb.org/gsea/msigdb/index.jsp), a public collection of functionally annotated gene sets, filtering for hallmark, curated (e.g., BioCarta, Broad Institute, Kyoto Encyclopedia of Genes and Genomes [KEGG]), and Gene Ontology (GO) gene sets in Homo Sapiens; 217 relevant gene sets were identified.

Each gene‐set was annotated to SNP positions on the  1k Genomes Project Phase 3 (  1kGP3) reference panel by using the Linkage Disequilibrium Score regression (LDSC) *make_annot.py* function (Bulik‐Sullivan et al., 2015). For the PRSs associated with SPs, the corresponding GWAS summary statistics were quality checked using the LDSC munging function, removing SNPs with a MAF ≤ 0.01 (when MAF was available) and those not matching HapMap3 SNPs, as these are generally well‐imputed; SNPs having missing or out‐of‐bounds values (ranges for INFO 0–1.5; MAF: 0–1; *p* values: 0–1), and all indels, structural and strand‐ambiguous variants were also pruned. Munged GWAS summary statistics of the phenotypic pairs under investigation were used as input datasets for the genetic covariance analyses. Significance levels were corrected for multiple comparisons by considering a maximum acceptable false discovery rate (FDR) of q = 0.05 (Benjamini & Hochberg, 1995).

In order to prioritize the results obtained in GNOVA, we performed partitioned heritability analyses in LDSC by assessing the $h_{\mathrm{SNP}}^{2}$ of SA at the level of the individual tested gene sets (Finucane et al., 2015). The same munged summary statistics used in the GNOVA analyses, as well as LD scores partitioned for each gene set were used as input in LDSC for these analyses.

## 3 RESULTS

After QC of target samples, a total of 3,834 patients were included in the PRS meta‐analyses for SA, of which 688 were cases [*N*_eff_ = 4/(1/*N* cases + 1/*N* controls) = 2,258]. Of these 3,834 subjects, 478 were diagnosed with schizophrenia, 2,601 with MDD, and 755 with bipolar spectrum disorders (Table 2).

**TABLE 2:** Number of individuals (after quality control steps) showing either SA or TWESI in the four clinical cohorts included in our analyses

	Non‐SA	SA	N	Age	Sex
				mean (*SD*) controls/cases	% males controls/cases
*CATIE*	455	23	478	41.00/41.15 (11.47/11.34) *t* = −0.13, *p* = .89	0.78/0.61 𝜒^2^ = 2.75, *p* = .10
*GSRD*	1,037	112	1,149	51.80/52.33 (13.93/15.48) *t* = −0.33, *p* = .74	0.33/0.44 𝜒^2^ = 4.89, *p* = .03*
STAR*D	1,241	211	1,452	43.96/38.22 (13.59/11.93) *t* = 6.32, *p* = 8.97 × 10^−10^ *	0.42/0.31 𝜒^2^ = 8.45, *p* = .004*
*STEP‐BD*	413	342	755	41.65/40.78 (12.69/12.06) *t* = 0.97, *p* = .33	0.48/0.39 𝜒^2^ = 6.44, *p* = 0.01*
*Total*	*3,146*	*688*	*3,834*		

	Non‐TWESI	TWESI	*N*	Age	Sex
				mean (*SD*) controls/cases	% males controls/cases
*GSRD*	1,020	129	1,149	52.10/49.77 (14.06/14.07) *t* = 1.76, *p* = .08	0.35/0.27 𝜒^2^ = 3.16, *p* = .08
STAR*D	1,340	85	1,425	42.59/45.10 (13.36/12.52) *t* = −1.34, *p* = .19	0.41/0.46 𝜒^2^ = 0.311, *p* = .58
*Total*	*2,360*	*214*	*2,574*		

Abbreviations: CATIE, Clinical Antipsychotic Trials of Intervention Effectiveness; GSRD, European Group for the Study of Resistant Depression; *N*, sample size; SA, suicide attempt; SD, standard deviations; STAR*D, Sequenced Treatment Alternatives to Relieve Depression; STEP‐BD, Systematic Treatment Enhancement Program for Bipolar Disorder; TWESI, treatment‐worsening/emergent suicidal ideation; 𝜒^2^, Pearson's Chi‐squared test statistic with Yates' continuity correction; *t*, Welch two sample *t*‐test statistic.

* *p* value < .05.

The PRS meta‐analyses on TWESI included a total of 2,574 patients with MDD, of whom 214 subjects were cases (*N*_eff_ = 785) (Table 2).

Further details on the number and demographic characteristics of cases and controls in the four samples are shown in Table 2.

### 3.1 Association between PRSs for MDD and SA or TWESI across samples

After Bonferroni correction, our meta‐analyses highlighted a positive association between MDD‐PRS and SA at P_T_ = 0.05 (OR = 1.24; 95% CI 1.11–1.38; *p* = 1.73 × 10^−4^; *R* ^2^ = 0.4%–1.6%; Table S3). The direction of the association effect between MDD‐PRS and SA was concordant across the four target samples, and no heterogeneity was detected (χ^2^_QE_ = 2.55, QE *p* = .47; I^2^ = 0%; Figure S1 shows a forest plot of the MDD‐PRS meta‐analysis on SA). The largest effect was found in CATIE, albeit with a large standard error, while the most significant effect was found in STAR*D, where all the tested P_T_ showed nominally significant associations (*p* = .03–2.5 × 10^−3^, *R* ^2^ = 0.5%–1.1%; Table S4).

**FIGURE 1:**
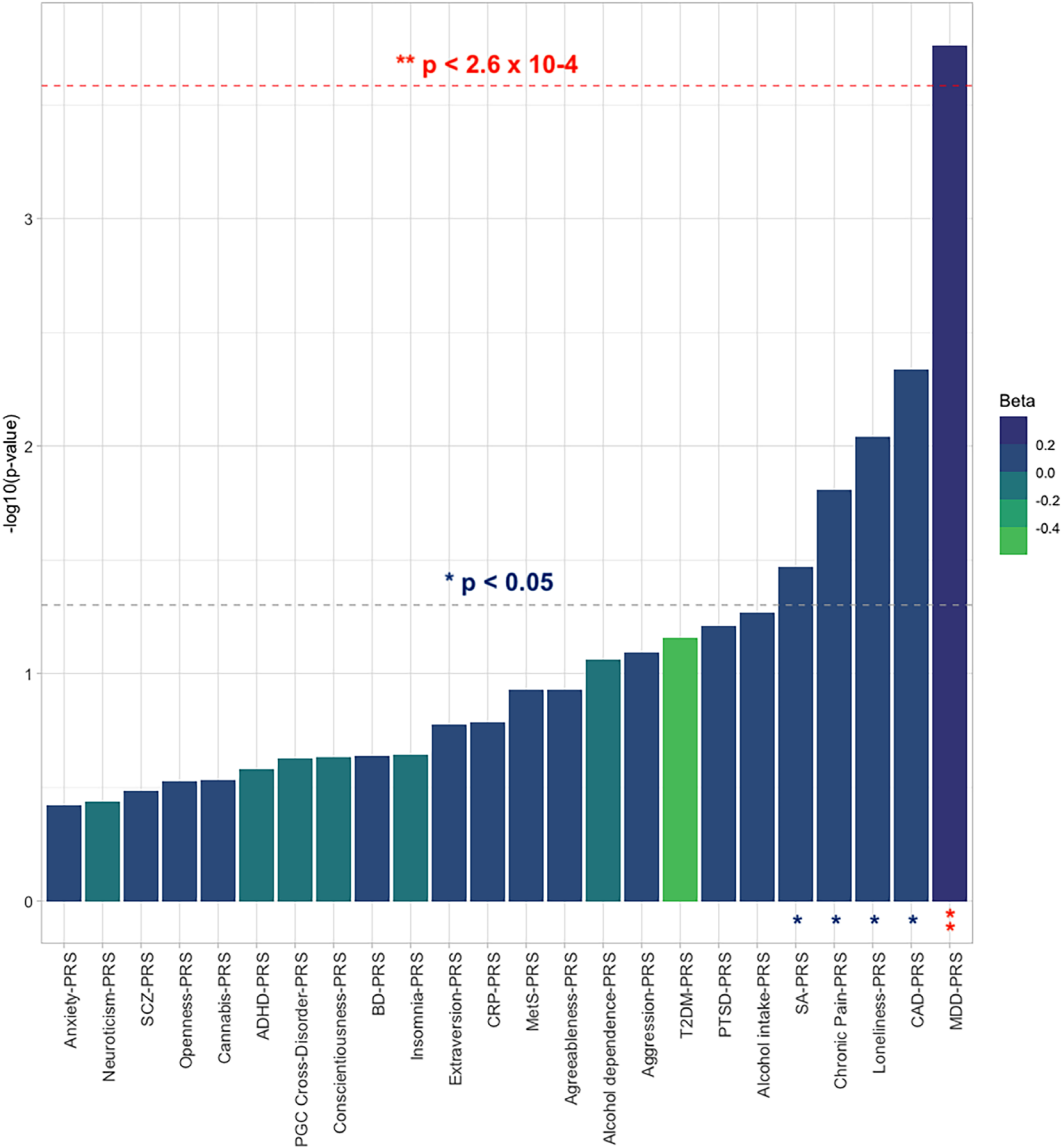
Bar plot showing the associations between the PRSs for multi neuropsychiatric, inflammatory, and cardio‐metabolic diseases/traits and suicide attempt (SA) in an overall case–control sample (after the meta‐analysis) of 3,834 individuals suffering from major depressive disorder, bipolar disorder spectrum or schizophrenia. Best‐fitting PRSs are depicted in increasing order of significance (−log10 p‐values) of association with SA. The red dashed line corresponds to the Bonferroni corrected threshold of statistical significance (α = .05/[24*8] = 2.6 × 10^−4^). The gray dashed line indicates the nominal threshold of significance (*p* = .05). ADHD, attention‐deficit/hyperactivity disorder; BD, bipolar disorder; CAD, coronary artery disease; CRP, C‐reactive protein; MDD, major depressive disorder; MetS, metabolic syndrome; PTSD, post‐traumatic stress disorder; T2DM, type 2 diabetes mellitus; SCZ, schizophrenia

**FIGURE 2:**
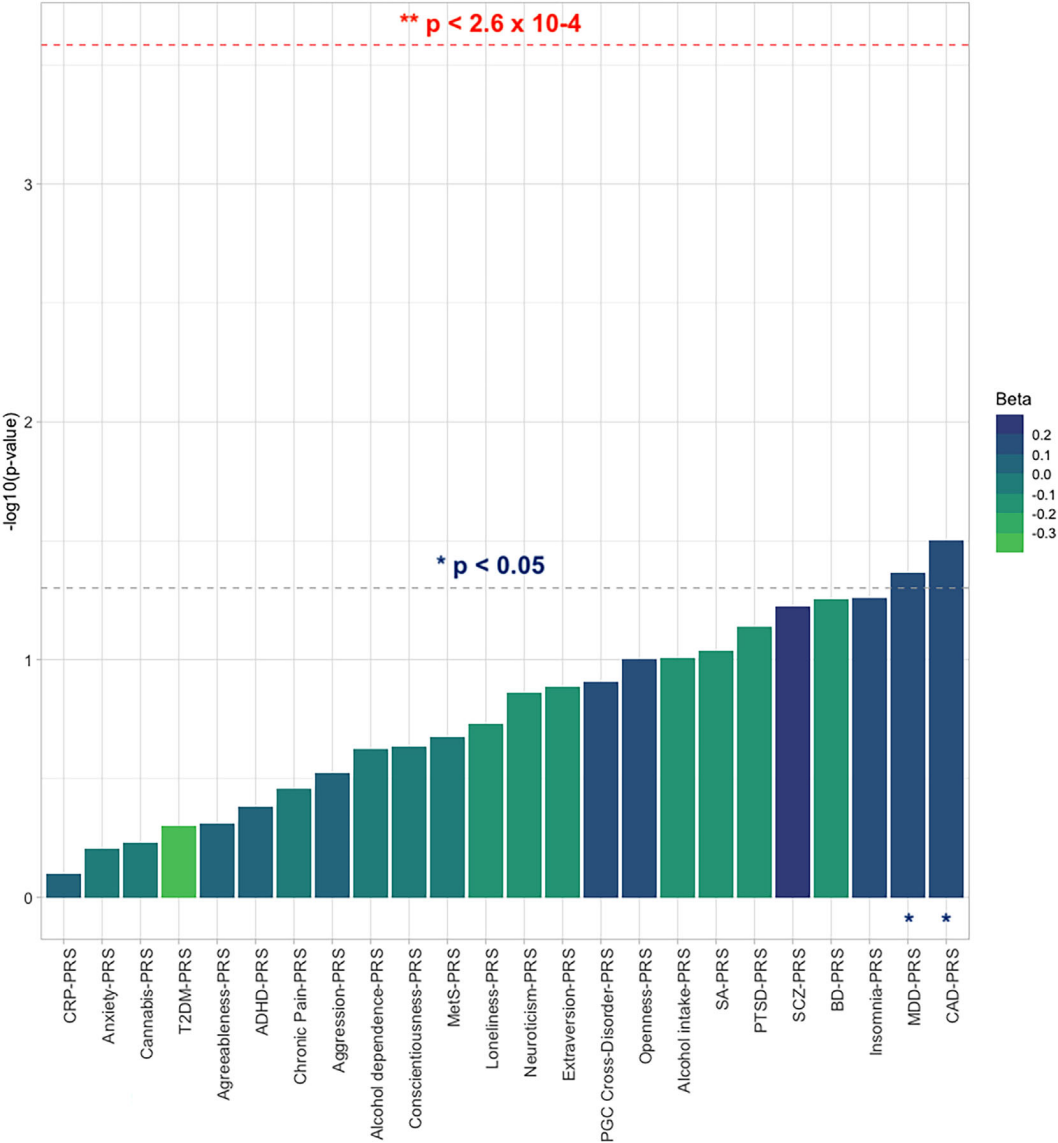
Bar plot showing the associations between the PRSs for multi neuropsychiatric, inflammatory, and cardio‐metabolic traits/diseases and the treatment‐emergent/worsening suicidal ideation (TWESI) phenotype in an overall case–control sample (after the meta‐analysis) of 2,574 individuals suffering from MDD. Best‐fitting PRSs are depicted in increasing order of significance (−log10 *p* values) of association with TWESI. The red dashed line corresponds to the Bonferroni corrected threshold of statistical significance (α = .05/[24*8] = 2.6 × 10^−4^). The gray dashed line indicates the nominal threshold of significance (*p* = .05). ADHD, attention‐deficit/hyperactivity disorder; BD, bipolar disorder; CAD, coronary artery disease; CRP, C‐reactive protein; MDD, major depressive disorder; MetS, metabolic syndrome; PTSD, post‐traumatic stress disorder; T2DM, type 2 diabetes mellitus; SCZ, schizophrenia

PRS meta‐analyses showed nominal associations between SA and the PRSs for CAD (*p* = 4.6 × 10^−3^), loneliness (*p* = .009), chronic pain (*p* = .016), and SA (*p* = .034) (Figure 1 and Table S3). No PRS was associated with TWESI after multiple‐testing correction, although MDD‐ and CAD‐PRS showed nominal associations in the meta‐analysis (*p* = .033 and *p* = .032, respectively) (Figure 2 and Table S5).

We also tested the relevance of age and sex as additional potential confounders, and the adjusted analyses found no substantial differences with the model including only recruitment centers and population PCs (data not shown).

### 3.2 Genetic covariance between MDD and SA stratified by gene sets

After FDR correction, functionally stratified GNOVA analyses revealed significant genetic covariance between MDD and SA at the level of 86 gene sets (Table S6), which were then prioritized by checking their partitioned SA $h_{\mathrm{SNP}}^{2}$. A significant $h_{\mathrm{SNP}}^{2}$ was found for the Reactome tumor necrosis factor receptor‐1 induced proapoptotic signaling (p_FDR_ = 0.007), GO α‐adrenergic receptor activity (p_FDR_ = 0.007), and GO adenylate cyclase inhibiting adrenergic receptor signaling pathway (p_FDR_ = 0.013) gene sets; however, only the $h_{\mathrm{SNP}}^{2}$ estimate for GO α‐adrenergic receptor activity had a value >0 ($h_{\mathrm{SNP}}^{2}$ = 8 × 10^−4^) (Table S6).

## 4 DISCUSSION

In this study, we investigated whether PRSs for 24 neuropsychiatric, inflammatory, and cardio‐metabolic diseases/traits were associated with SA or TWESI in a pooled sample of up to 3,834 patients with mood disorders or schizophrenia. Our results suggested that a higher genetic liability for MDD underlies an increased risk of SA among patients with psychiatric disorders. PRSs for loneliness, chronic pain, and SA were nominally associated with SA across samples, as well as MDD‐PRS with TWESI in patients with MDD. This was the first study to investigate whether the PRSs for inflammatory and cardio‐metabolic traits/diseases are associated with SA or TWESI. According to our findings, the impact of the genetic risk for inflammatory and cardio‐metabolic diseases/traits is absent or limited for the examined SPs.

Our findings are consistent with previous evidence of shared genetics between MDD and SA, as other authors have previously suggested through PRSs and bivariate genetic correlation analyses (*r*_g_ = .44) (Mullins et al., 2019; Ruderfer et al., 2020). An association between MDD‐PRS and SA had been demonstrated in patients with MDD, BD, and schizophrenia (*p* = 2 × 10^−4^ to 6 × 10^−4^; *R* ^2^ = .24–.40%) by using a base dataset for MDD approximately three times smaller (*n* = ~171,000) and independent target samples than those used in our analyses (Mullins et al., 2019). Of note, our results also showed a more significant effect of MDD‐PRS on SA in patients with MDD than in those affected by schizophrenia or bipolar spectrum disorders, although the association signal remained concordant across the four considered target samples. The genetic overlap with MDD may not be confined to SA, but also to TWESI, in line with previous studies (Mullins et al., 2014), though the association we found between MDD‐PRS and TWESI did not survive Bonferroni correction.

We further investigated the genetic overlap between MDD and SA using GNOVA and 217 gene sets relevant for the biological mechanisms involved in suicide; the results showed significant genetic covariance at the level of 86 gene sets. Among these, the majority (i.e., 70/86) showed a concordant direction of effect between MDD and SA, as expected from the PRS analyses and previous epidemiological evidence. For the gene sets showing significant genetic covariance, the partitioned $h_{\mathrm{SNP}}^{2}$ of SA supported a positive and significant genetic component only for the α‐adrenergic receptor activity gene set (GO:0004936). α_2_‐adrenoreceptors are mostly auto−/hetero‐receptors located at the presynaptic level and negatively regulate the release of neurotransmitters such as norepinephrine and serotonin (Aoki, Venkatesan, Go, Forman, & Kurose, 1998). Replicated evidence demonstrated an up‐regulation and increased activity of α_2_‐adrenoreceptors in the hippocampus and prefrontal cortex of MDD patients who died by suicide; in these individuals, α_2_‐adrenoreceptors seem not affected by the expected down‐regulatory effects of antidepressants (Rivero et al., 2014). Interestingly, noradrenergic neurotransmission also plays a role in aggression, which is considered an endophenotype of suicide, and higher plasma levels of norepinephrine have been associated with decreased aggressive and suicidal behaviors (Yanowitch & Coccaro, 2011). The role of α_2_‐adrenoreceptors in suicide is also supported by the observation that clozapine has the greatest differential affinity toward α‐adrenoreceptors versus D_2_‐receptors (the most potent affinity with respect to binding to the dopamine D_2_‐receptor) among atypical antipsychotics (Stahl & Stahl, 2013). The lack of a positive and significant $h_{\mathrm{SNP}}^{2}$ for SA in the other gene sets showing significant genetic covariance between SA and MDD may reflect a truly nonsignificant genetic component for SA in those pathways, but also be the consequence of sampling variation in small samples when the true heritability is near zero; in this regard, limited statistical power was suggested by previous GWAS of SA, since the large discrepancy between $h_{\mathrm{SNP}}^{2}$ (4%) and heritability found by twin studies (30%–50%).

Among the nominally significant findings, we report that loneliness‐PRS was associated with SA in the meta‐analysis, and a more significant effect was found in MDD. This finding appears relevant as loneliness is one of the core symptoms of depression, and it has been longitudinally associated with SA (Gijzen et al., 2021; Solmi et al., 2020). We cannot exclude that the effect of the genetic risk of loneliness on SA is indirectly mediated by the genetic overlap of loneliness and SA with MDD. However, while loneliness and MDD are genetically correlated (*r*_g_ = .61), it has been shown that the genetic risk loci for loneliness are independent of susceptibility for depression, suggesting that depression and loneliness are at least in part distinct conditions (Day, Ong, & Perry, 2018).

We also reported a nominal association between CAD‐PRS and both SA and TWESI, in line with the large amount of clinical evidence pointing to an increased risk of suicide in people with cardiovascular disease and vice versa (Artero, Astruc, Courtet, & Ritchie, 2006; Zhong et al., 2020). While MDD has also been shown to be a risk factor for and genetically correlated with CAD (*r*_g_ = .12) (Wray et al., 2018), the association between CAD and SPs may be independent of depression risk, as suggested by previous studies (Artero et al., 2006; Moazzami et al., 2018).

Our study comes with some strengths and limitations. The major strengths are the use of a panel of 24 base traits/diseases and individual‐level genotypes from four trans‐diagnostic target samples for PRS analyses. Other strengths were the use of PRS meta‐analyses to summarize the results across samples and a strict Bonferroni correction that minimized type‐1 errors. Our analyses were limited to neuropsychiatric, inflammatory, and cardio‐metabolic traits/diseases and did not consider other medical (e.g., respiratory, oncological) conditions either because these were limited in power, have greater phenotypic and genetic heterogeneity, or a lower polygenic component (Burrell, McGranahan, Bartek, & Swanton, 2013). A further asset is the study of the neurobiology underlying the association between MDD‐PRS and SA through a genetic covariance analysis stratified by gene sets related to well‐established antisuicidal drugs. We are aware that other molecules have been indicated as possible mitigators of suicidality (e.g., omega‐3‐fatty acids, buprenorphine), but the related evidence is weaker and needs replication before being used for such genomic studies (Pompili et al., 2017; Turecki et al., 2019). Some other limitations did not allow clear conclusions to be drawn. Among them, the small effective sample size (*N*_eff_, with a much lower number of cases than controls) and $h_{\mathrm{SNP}}^{2}$ explained in the SA GWAS used as reference for our GNOVA analyses (Ruderfer et al., 2020), which made us cautious in interpreting the GNOVA results. Hence, these findings are currently to be considered as exploratory. There was residual overlap between the base and target samples for BD‐PRS analyses on SA; however, the extent of the overlap was minimal, constituting 19.7% and 0.18% of the target and base sample, respectively.

In conclusion, our study highlighted that a higher genetic liability for MDD increases SA risk among patients with mood disorders and schizophrenia, pointing to possible shared etiopathogenetic mechanisms between MDD and SA. In this regard, we suggested a convergent genetic signal at the level of the α‐adrenoreceptor signaling pathway, in line with previous evidence linking this pathway with both MDD and suicide. Overall, our findings suggested limited or no genetic overlap between inflammatory and cardio‐metabolic traits and SPs. Despite only nominally significant, the association between loneliness‐PRS and SA is consistent with previous strong evidence supporting the relevance of this trait on suicide risk. Therefore, the early and proper treatment of MDD, with a particular focus on feelings of loneliness, should be considered pivotal to reducing suicide rates. These results may be useful for implementing algorithms that incorporate both clinical and genetic risk factors to identify individuals at higher risk for SA and thus eligible for targeted prevention campaigns.

## CONFLICT OF INTEREST

Siegfried Kasper received grants/research support, consulting fees and/or honoraria within the last 3 years from Angelini, AOP Orphan Pharmaceuticals AG, AstraZeneca, Eli Lilly, Janssen, KRKA‐Pharma, Lundbeck, Neuraxpharm, Pfizer, Pierre Fabre, Schwabe, and Servier. Julien Mendlewicz is a member of the board of the Lundbeck International Neuroscience Foundation and of the advisory board of Servier. Stuart Montgomery has been a consultant or served on advisory boards for AstraZeneca, Bionevia, Bristol‐Myers Squibb, Forest, GlaxoSmithKline, Grunenthal, Intellect Pharma, Johnson & Johnson, Lilly, Lundbeck, Merck, Merz, M's Science, Neurim, Otsuka, Pierre Fabre, Pfizer, Pharmaneuroboost, Richter, Roche, Sanofi, Sepracor, Servier, Shire, Synosis, Takeda, Theracos, Targacept, Transcept, UBC, Xytis, and Wyeth. Alessandro Serretti is or has been a consultant/speaker for Abbott, Abbvie, Angelini, AstraZeneca, Clinical Data, Boehringer, Bristol‐Myers Squibb, Eli Lilly, GlaxoSmithKline, Innovapharma, Italfarmaco, Janssen, Lundbeck, Naurex, Pfizer, Polifarma, Sanofi, and Servier. Daniel Souery has received grant/research support from GlaxoSmithKline and Lundbeck, and he has served as a consultant or on advisory boards for AstraZeneca, Bristol‐Myers Squibb, Eli Lilly, Janssen, and Lundbeck. Josep Zohar has received grant/research support from Lundbeck, Servier, and Pfizer; he has served as a consultant on the advisory boards for Servier, Pfizer, Solvay, and Actelion; and he has served on speakers’ bureaus for Lundbeck, GSK, Jazz, and Solvay. The other authors declare no conflict of interest.

## ETHICS STATEMENT

Data were obtained for analysis from the National Institute of Mental Health (NIMH), Bethesda, Maryland, US (Request ID 5ce26a95712d8). The STAR‐D, STEP‐BD, and CATIE trials were conducted according to the Principles of Helsinki Declaration. The study protocol was reviewed and approved by ethical committees at local recruitment sites. All subjects selected by clinicians were included in the screening phase after obtaining their written informed consent. This research group certifies that data collected for the STAR‐D, STEP‐BD, and CATIE trials were exclusively used for scientific investigation. Before obtaining access to data, the objectives of our investigation were clearly described in the request form.

## Supporting information


**Figure S1** Forest plot showing the association between the polygenic risk score for major depressive disorder (calculated at the genome‐wide P‐threshold of 0.05) and suicide attempt across the four included clinical cohorts.

**Table S1** Results of the genetic covariance analyses between major depressive disorder (MDD) and suicide attempt (SA) stratified by gene sets related to clozapine, ketamine, lithium effect.

## Data Availability

Target datasets for this publication were partly obtained from NIMH Repository & Genomics Resource, a centralized national biorepository for genetic studies of psychiatric disorders.
