# Supplementary material for: Polygenic risk scores for neuropsychiatric, inflammatory, and cardio‐metabolic traits highlight possible genetic overlap with suicide attempt and treatment‐emergent suicidal ideation
Source: Am J Med Genet B Neuropsychiatr Genet. 2022 Feb 21;189(3-4):74–85. doi: 10.1002/ajmg.b.32891 (PMC9305542; doi:10.1002/ajmg.b.32891)
Supplement: Supplementary file 1 — Figure S1 Forest plot showing the association between the polygenic risk score for major depressive disorder (calculated at the genome‐wide P‐threshold of 0.05) and suicide attempt across the four included clinical cohorts. [file AJMG-189-74-s001.docx]

Table of Contents

[1. Extraction of the target phenotypes for polygenic risk score analyses 2](#_Toc83831423)

[2. Genotyping of the target samples for polygenic risk score analyses 2](#_Toc83831424)

[Figure S1 4](#_Toc83831425)

[References 5](#_Toc83831426)

# **Extraction of the target phenotypes for polygenic risk score analyses**

In the Clinical Antipsychotic Trials of Intervention Effectiveness (CATIE) study, lifetime suicide attempt (SA) was assessed based on the Calgary Depression Rating Scale (CDRS) item 8 (“Suicide” – points 4: “Suicidal attempt…"), the Family/Caregiver Interview (FI) item 78 (“Attempted suicide during the past 6 months”) at baseline and follow-up visits. In the European Group for the Study of Resistant Depression (GSRD) sample, SA was defined according to the HDRS (Hamilton Depression Rating Scale) item 3 (“Suicide” – points 4: “Suicide attempts”) or MINI (Mini-International Neuropsychiatric Interview) items C5 and C6a (“In the past month did you attempt suicide?” and “In your lifetime did you ever make a suicide attempt?”, respectively). In the Sequenced Treatment Alternatives to Relieve Depression (STAR*D) study, SA was assessed through the HDRS item 3 (“Suicide” – points 4: “Suicide attempts”), the screening CRF (Case Report Form) item 21 (“Attempted suicide”), SAE (Serious Adverse Event) form item 19 (“Failed suicide attempt”), IVR (Interactive Voice Response) Alert form (“Primary reason for alert: suicide attempt”). In the, Systematic Treatment Enhancement Program for Bipolar Disorder (STEP-BD) study, SA was derived from the SQ (Suicide Questionnaire), combining item 3 (“Tried to hurt yourself or attempt suicide?”) and item 5 (“Did you intend to die?”), SAE (Serious Adverse Experience) report ("Was there a suicide attempt?"), ADE (Affective Disorders Evaluation) form (“History of suicide attempts”), Care Utilization (CU) form (“Suicide attempts, last 3 months”).

In line with the previous literature [Laje and others 2007; Perlis and others 2007], treatment-worsening/emergent suicidal ideation (TWESI) was derived in GSRD as a Δ≥1 change from baseline in MADRS (Montgomery Asberg Depression Rating Scale) item 10 score and in STAR*D as a Δ≥1 change from baseline in the QIDS-SR16 (16-item Quick Inventory of Depressive Symptomatology – Self-Report) item 12.

# **Genotyping of the target samples for polygenic risk score analyses**

Individuals in the CATIE study were genotyped by Perlegen Sciences (Mountain View, CA, USA) using the Affymetrix GeneChip Human Mapping 500K Array Set (Santa Clara, CA, USA), comprising Nsp and Sty chips, and a custom 164K chip built by Perlegen to obtain a wider genome coverage. Genotyping of the GSRD participants was conducted using the Illumina Infinium PsychArray-24 BeadChip (San Diego, CA, USA). Individual-level genotypes in STAR*D were obtained using the Affymetrix GeneChip Human Mapping 500K Array Set or Affymetrix Genome-Wide Human SNP Array 5.0. For STEP-BD, genotyping was carried out by the Genetic Analysis Platform at the Broad Institute (Cambridge, MA, USA) using the Affymetrix GeneChip Human Mapping 500K Array Set.

**Figure S1**. **Forest plot showing the association between the polygenic risk score for major depressive disorder (calculated at the genome-wide P-threshold of 0.05) and suicide attempt across the four included clinical cohorts.**

Abbreviations: FE, fixed effects; CI, confidence intervals; OR, Odds Ratio; CATIE, Clinical Antipsychotic Trials of Intervention Effectiveness; GSRD, European Group for the Study of Resistant Depression; STAR*D, Sequenced Treatment Alternatives to Relieve Depression, STEP-BD, Systematic Treatment Enhancement Program for Bipolar Disorder.


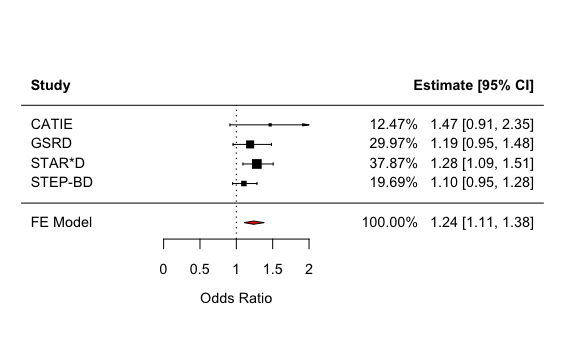


Test for overall effect: **OR = 1.24; 95% CI = 1.11-1.38; p = 1.73 x 10^-4^**

# **References**

Laje G, Paddock S, Manji H, Rush AJ, Wilson AF, Charney D, McMahon FJ. 2007. Genetic markers of suicidal ideation emerging during citalopram treatment of major depression. Am J Psychiatry 164(10):1530-1538.

Perlis RH, Purcell S, Fava M, Fagerness J, Rush AJ, Trivedi MH, Smoller JW. 2007. Association between treatment-emergent suicidal ideation with citalopram and polymorphisms near cyclic adenosine monophosphate response element binding protein in the STAR*D study. Arch Gen Psychiatry 64(6):689-697.
